# Association of the Red Complex Bacteria in Endo-Perio Cases Before and After Endo-Perio Therapy: A Clinico-Microbiological Study

**DOI:** 10.7759/cureus.103444

**Published:** 2026-02-11

**Authors:** Dilip Goswami, Reshu Rai, Mhayani Jami, Prachi Jain, Parivraj Priyam Goswami

**Affiliations:** 1 Periodontics and Oral Implantology, Regional Dental College, Guwahati, IND; 2 Medicine, Saveetha Medical College, Chennai, IND

**Keywords:** bana test, endodontic therapy, endo-perio lesion, periodontal therapy, red complex

## Abstract

Introduction: Pulp and periodontal diseases are among the most frequent causes of tooth loss. Due to the anatomical continuum between the pulp and the periodontium, pathological shifts in one tissue frequently cause a secondary response in the other, manifesting as a combined endodontic-periodontal lesion. Treating endo-perio cases requires a systematic, staged, combined approach that addresses both the endodontic and periodontal components of the lesion. Traditional success criteria rely on clinical and radiographic assessments; however, these physical measurements do not always reflect the ongoing biological status of the disease. Comprehensive evaluation of therapeutic outcomes requires the integration of microbiological data and molecular biomarkers, which serve as critical indices for monitoring current disease activity and predicting long-term clinical stability. The strategic implementation of microbial assays at follow-up stages can help the clinician to improve the management of the case, ensuring that interventions are personalized to the individual case.

Objectives: The objective of this study is to evaluate the presence of "red complex" bacteria (*Porphyromonas gingivalis*,* Treponema denticola*, and *Tannerella forsythia*) in endo-perio lesions and to compare the effect of endodontic therapy alone and the combined approach of endodontic and periodontal therapies on the levels of these bacteria.

Materials and methods: This prospective clinico-microbiological study involved 40 research participants of the age group (20-60 years), all diagnosed with primary periodontal lesions with secondary endodontic involvement as per Simon’s classification (1972). Participants were randomly assigned to two treatment groups: Group A (n = 20) received endodontic treatment only. Group B (n = 20) received endodontic treatment followed by periodontal treatment at three months. N-benzoyl-DL-arginine-2-napthylamide-enzymatic™ (BANA) test and clinical parameters (gingival index (GI), probing pocket depth (PPD) and clinical attachment level (CAL)) were assessed at baseline, three months, and six months.

Results: Before treatment, 75% of research participants were BANA-positive. After treatment, there was a significant reduction of red complex bacteria from baseline to six months (p < 0.001) in both groups. The Chi-square analysis showed no significant difference in the distribution of BANA status between the groups at three months (p = 0.692) and six months (p = 0.942). Both groups demonstrated an improvement of clinical parameters such as GI, PPD and CAL from baseline to six months (p < 0.05). However, the F-statistic for overall study progress was higher in Group B (F = 33.682) compared to Group A (F = 26.845).

Conclusion: The combined therapeutic approach (Group B) results in a more statistically significant reduction in the red complex bacteria.

## Introduction

The pulp and periodontium are closely linked through their embryonic origins, anatomical structures, and physiological functions. The anatomical pathways, such as the apical foramen, lateral and accessory canals, and dentinal tubules, facilitate the bidirectional nature of infection and inflammation spread, emphasizing the need for a comprehensive understanding of their complex relationship. The pathological processes affecting one tissue often affect the other, resulting in the complex clinical condition known as the endodontic-periodontal lesion [1-6].

The "red complex," which includes *Porphyromonas gingivalis*, *Treponema denticola*, and *Tannerella forsythia *(formerly *Bacteroides forsythus*), encompasses the most important pathogens in periodontal disease [7,8]. Current understanding suggests that the pulp and periodontium are not an isolated system. Thus, these periodontal pathogens can influence the endodontic environment as well, further complicating the clinical presentation of the disease [9-11]. Simon et al. (1972) emphasized the classification of endo-perio lesions for accurate diagnosis and for determining an appropriate therapeutic approach necessary to address both the pulpal and periodontal components [12]. This cross-contamination suggests that treating one system in isolation may be insufficient when pathogens have migrated between these distinct anatomical sites [13,14].

The basis of endo-perio therapy lies in a combined approach that addresses both the endodontic and periodontal components of the lesion [15-17]. Following treatment, success is assessed by the reduction in clinical signs and symptoms. Clinical assessments, such as bleeding on probing, probing depth, attachment level, and radiographic assessment, are essential for evaluating treatment success [18-20]. However, they often fail to reflect the underlying biological activity of the disease. A comprehensive understanding of treatment efficacy needs the integration of microbiological assessments that can provide insights into the microbial burden and potential for future disease progression [21,22].

A comprehensive investigation specifically focusing on the longitudinal changes in these key pathogens before and after endodontic-periodontal therapy remains limited. Therefore, this study aims to address the gap by employing the BANA test as a semiquantitative measure of red complex bacteria in endo-perio lesions [23-25]. This investigation will provide a comparative analysis of the impact of endodontic therapy alone and the combined approach of endodontic and periodontal therapies on the levels of these pathogenic bacteria, along with the evaluation of clinical parameters [26-28].

BANA enzymatic™ test analysis

The BANA-Zyme™ test (N-benzoyl-DL-arginine-2-naphthylamide) is a rapid chairside diagnostic tool used to detect specific anaerobic bacteria. The "red complex" pathogens, *P. gingivalis*, *T. denticola*, and *T. forsythia*, possess a unique protease capable of hydrolyzing the synthetic peptide analog, BANA (N-benzoyl-DL-arginine-2-naphthylamide). The test utilizes a reagent strip consisting of two distinct matrices affixed to a plastic card: (1) a lower matrix, impregnated with the BANA substrate, where the subgingival plaque sample is applied; and (2) an upper matrix, which contains a chromogenic diazo reagent (Fast Black K). When the specific peptidase is present in the sample, it hydrolyzes the BANA substrate. The resulting breakdown product then reacts with the Fast Black K in the upper matrix, producing a permanent blue color. This color change indicates a positive or weak-positive result. While blood and saliva do not hydrolyze BANA or interfere with the chemical reaction, the presence of blood in a sample may physically obscure the visualization of the blue color.

## Materials and methods

This was a one-year prospective comparative study conducted from March 10, 2024, to March 10, 2025, at the Regional Dental College, Guwahati, India. Initial approval for the study was obtained from the Institutional Ethics Committee of Regional Dental College, Guwahati, India (RDC/29/2011/2694). The sample size was determined using G*Power software, version 3.1.9.7 (Heinrich-Heine-Universität Düsseldorf, Düsseldorf, Germany), targeting a medium-to-large effect size (d = 0.90) based on previous endo-perio literature. With an alpha level of 0.05 and 80% statistical power, the analysis required a minimum of 32 participants, divided into two treatment groups. To account for a probable 20% attrition rate, the recruitment target was increased to 40 participants (20 per group). This adjustment ensured the final analysis retained sufficient power to identify statistically significant differences between the treatment modalities, even after excluding cases lost to follow-up.

Inclusion criteria

The following inclusion criteria were applied in the study: patients aged 20-60 years with a periodontal pocket depth of ≥5 mm at one or more sites on the affected tooth, radiographic evidence of periodontal ligament (PDL) widening and alveolar bone loss adjacent to the affected tooth, clinical attachment loss (CAL) at one or more sites on the affected tooth, and clinical and/or radiographic evidence of pulpal involvement (e.g., periapical radiolucency, history of pain, tenderness to percussion). Patients presented with a primary periodontal lesion with secondary endodontic involvement according to Simon’s classification (1972) [12].

Exclusion criteria

The following exclusion criteria were applied in the study: diagnosis of aggressive periodontitis, history of antibiotic or anti-inflammatory drug intake within the preceding three months, presence of known systemic diseases or conditions that could affect periodontal or endodontic treatment outcomes (e.g., uncontrolled diabetes and immunocompromised status), pregnant women and lactating mothers, teeth with vertical root fractures or extensive non-restorable coronal destruction, and participants unwilling or unable to comply with the study protocol and follow-up appointments.

A total of 40 research participants presenting with endo-perio lesions (22 males, 18 females, aged 20-59 years, comprising 16 molars, five premolars, three canines, and 16 incisors) were included into the study. All the research participants received a detailed explanation of the study objectives, treatment procedures, potential risks, and benefits. Informed written consent was obtained from each participant before their enrolment in the study. The randomization was performed sequentially by the concealed enveloped technique upon enrolment to ensure concealment of allocation. The allocation sequence was managed by an independent researcher who was not involved in the clinical procedures, and the researcher performing the clinical parameter measurements and the BANA test analysis were also blinded to the treatment allocation of the participants. Statistical analysis was performed by a statistician blinded to the group assignments.

Procedure

Endodontic Treatment

Following clinical diagnosis, endodontic therapy was initiated with straight-line access preparation. Apical patency was established using size #10 or #15 stainless steel K-files (Dentsply Sirona, Switzerland). The working length was initially determined using an electronic apex locator (Root ZX II; J. Morita Corp., Kyoto, Japan) and subsequently verified via digital intraoral periapical radiographs. Biomechanical preparation was performed using a combination of ProTaper Gold hand and rotary files (Dentsply Sirona, Charlotte, NC) utilized in a crown-down sequence. Throughout instrumentation, the canals were irrigated with 5.25% sodium hypochlorite (NaOCl) (Septodont, France) and 0.9% sterile saline (Baxter India Pvt. Ltd., Gurgaon, Haryana, India) using a 30-gauge side-vented irrigation needle (Max-i-Probe; Dentsply Sirona, Charlotte, NC). Between appointments, an aqueous calcium hydroxide paste (ApexCal; Ivoclar Vivadent, Amherst, NY) was placed as an intracanal medicament for 7-10 days. The canals were subsequently obturated with ProTaper Gold conformational gutta-percha points (Dentsply Sirona, Charlotte, NC) and a resin-based root canal sealer (AH Plus; Dentsply, Germany). Following obturation, access cavities were permanently restored with a composite resin, and prosthetic crowns were fabricated and placed where clinically indicated.

Periodontal Treatment

The extraoral surgical site was disinfected using a 10% povidone-iodine antiseptic solution (Betadine; Win-Medicare Pvt. Ltd., New Delhi, India). Local anesthesia was achieved via infiltration using 2% lidocaine hydrochloride with 1:80,000 epinephrine (Lignox; Warren Pharmaceuticals Ltd., Mumbai, India) administered with a 30-gauge disposable needle (Septoject; Septodont, Saint-Maur-des-Fossés, France). A crevicular incision was performed using a No. 15 stainless steel surgical blade (Swann-Morton Ltd., Sheffield, South Yorkshire, England) attached to a No. 3 stainless steel scalpel handle. Full-thickness mucoperiosteal flaps were elevated using a Periosteal Elevator (#24G; Hu-Friedy, Chicago, IL) to provide comprehensive visualization of the underlying osseous defects. The incision was designed specifically to preserve marginal gingival integrity, ensuring tension-free primary closure. Granulation tissue on the internal aspect of the reflected flaps was meticulously debrided using Gracey curettes (Hu-Friedy, Chicago, IL). Root surfaces were subjected to thorough scaling and root planing (SRP) using ultrasonic inserts (P-10; Hu-Friedy, Chicago, IL) and hand instruments until a smooth, hard consistency was achieved. The surgical field was copiously irrigated with 0.9% sterile saline (Baxter India Pvt. Ltd., Gurgaon, Haryana, India). The buccal and lingual flaps were repositioned and secured using 4-0 non-resorbable black braided polyamide (nylon) sutures (Ethilon; Ethicon, Johnson & Johnson, Somerville, NJ) in an interrupted pattern. A non-eugenol periodontal dressing (Coe-Pak; GC America, Alsip, IL) was applied over the surgical site to protect the wound and stabilize the flaps. Standardized postoperative instructions were provided, and sutures were removed seven days post-surgery.

Data collection

**Figure 1:**
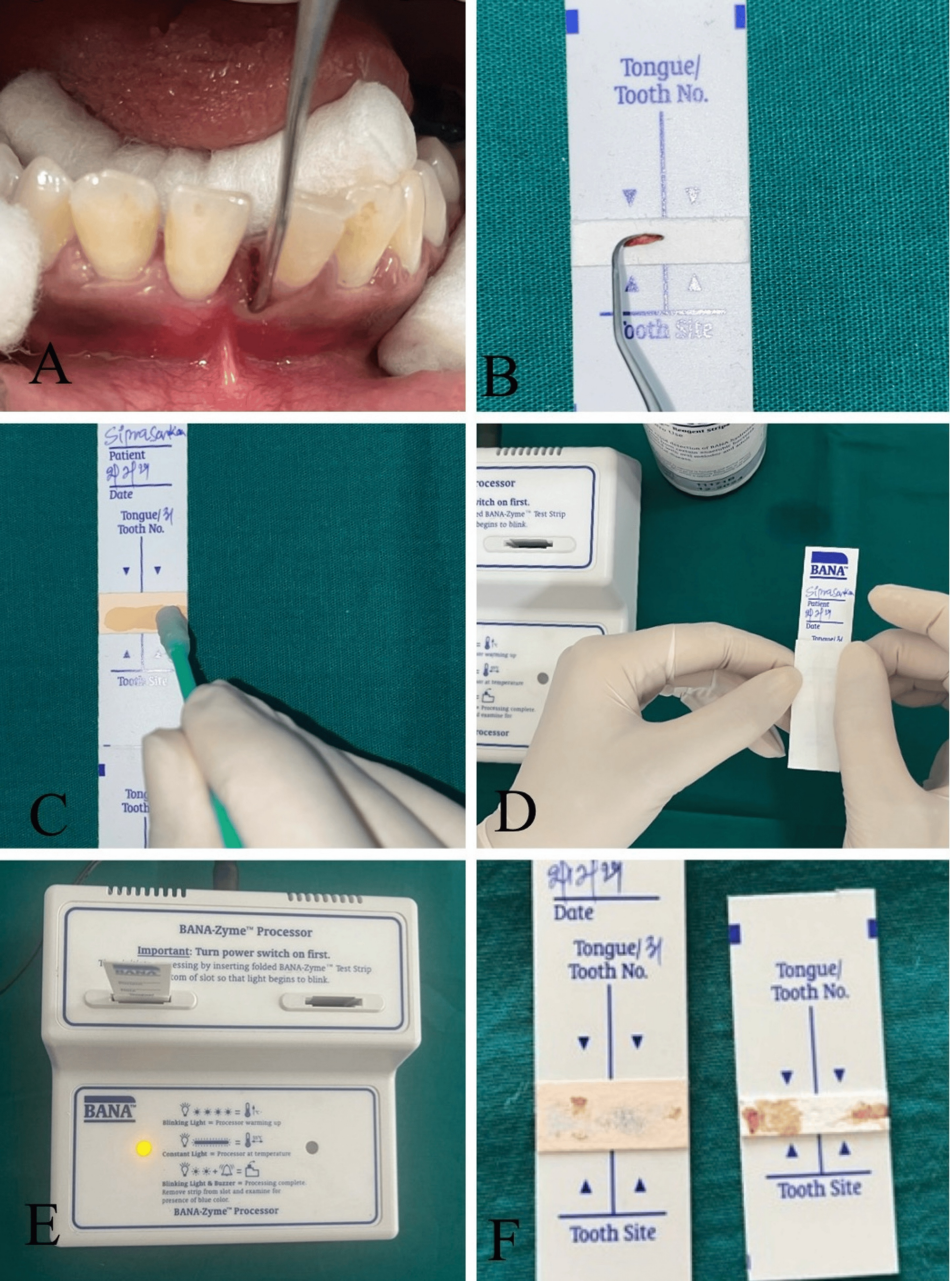
Procedural steps for the BANA-Zyme™ test, including (A) subgingival plaque sampling, (B-D) sample application and strip preparation, (E) incubation in the processor, and (F) final visual comparison of results

Microbiological analysis was performed using the BANA-Zyme™ test with the BANA test kit (Figure 1).

**Figure 2:**
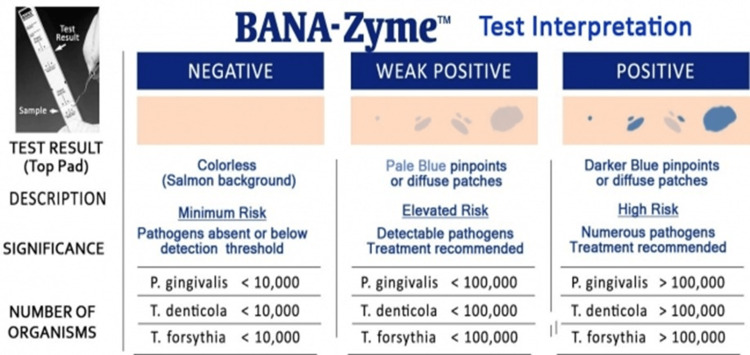
Interpretation guide for the BANA-Zyme™ test results, illustrating the colorimetric scale from negative (colorless/salmon) to positive (dark blue) based on the concentration of "red complex" pathogens

The BANA-Zyme™ enzymatic assay was performed according to the manufacturer’s instructions (Ora Tec Corp., Manassas, VA) using the following standardized protocol: prior to sample collection, supragingival plaque was removed. Subgingival plaque was then collected from the deepest portion of the periodontal pocket using a sterile Gracey curette (Hu-Friedy, Chicago, IL). The collected plaque was applied directly onto the lower reagent matrix of the BANA-Zyme™ strip. To prevent cross-contamination between sites, the curette was meticulously wiped with a clean, sterile gauze pad after each application. Once all samples were secured, the upper reagent matrix (salmon-colored pad) was lightly moistened with distilled water using a sterile cotton tip applicator to activate the chromogenic reaction. The strip was folded at the perforated crease, ensuring direct contact between the upper and lower matrices. The assembly was then inserted into a calibrated BANA-Zyme™ Processor (Ora Tec Corp., Manassas, VA) and incubated for 15 minutes at 55℃ ± 5℃.The reaction was considered complete upon the auditory signal (bell) of the processor. Result interpretation was done using interpretation guide (Figure 2).

Recording of clinical parameters

The clinical periodontal status of each participant was evaluated by a single calibrated examiner. Gingival health was quantified using the gingival index (GI) as established by Löe and Silness (1963), where a score of 0 denotes a healthy clinical state with no inflammation; a score of 1 indicates mild inflammation characterized by subtle color changes and incipient edema without bleeding on probing; a score of 2 signifies moderate inflammation with manifest erythema, glazing, and bleeding upon probing; and a score of 3 represents severe inflammation marked by pronounced redness, hypertrophy, ulceration, and a tendency toward spontaneous hemorrhage. Concurrent with the GI assessment, probing pocket depth (PPD) and CAL were recorded at four specific sites per tooth: the distofacial, midfacial, and mesiofacial line angles, and the center of the lingual surface. All measurements were obtained using a standardized UNC-15 periodontal probe (Hu-Friedy Mfg., Chicago, IL).

Statistical analysis

Data were compiled and managed using Microsoft Excel 2009 (Microsoft Corp., Redmond, WA). Statistical analyses were performed using IBM SPSS Statistics for Windows, version 20.0 (IBM Corp., Armonk, NY). The Shapiro-Wilk test was initially utilized to assess the normality of the data distribution. For continuous variables, including GI, PPD, and CAL, intragroup comparisons (baseline to six months) were analyzed using the student’s paired t-test. Intergroup comparisons between Group A and Group B were conducted using the independent samples t-test. For categorical data involving the BANA enzymatic test results, the Chi-square test was employed for intergroup comparisons of microbial presence. The Wilcoxon signed-rank test was used to analyze intragroup shifts in BANA scores (positive, weak positive, and negative) over the follow-up intervals. Statistical significance for all tests was predetermined at a p-value < 0.05 at a 95% confidence interval.

## Results

The demographic and baseline characteristics of research participants are given in Table 1.

**Table 1:** The demographic and baseline characteristics of research participants Age: Data presented as mean ± standard deviation. Tooth type (I:C:P:M): Distribution of the involved teeth categorized as I (incisors), C (canines), P (premolars), and M (molars). BANA: N-benzoyl-DL-arginine-2-naphthylamide.

Characteristics	Group A (n = 20)	Group B (n = 20)
Age (years)	41.5 ± 12.4 (20–59)	41.8 ± 10.3 (30–55)
Gender (M:F)	11:9 (55%:45%)	8:12 (40%:60%)
Tooth type (I:C:P:M)	8:0:3:9	8:2:3:7
BANA test analysis	Positive: 15 (75%); Weak positive: 5 (25%)	Positive: 15 (75%); Weak positive: 5 (25%)

Microbiological analysis (BANA test)

The BANA test results reflected a shift in the subgingival microflora for both groups.

Baseline Status

A high proportion of red complex bacteria was observed, with 80% of Group A and 75% of Group B testing BANA-positive for "red complex" bacteria. There was a significant reduction of red complex bacteria from baseline to six months (p < 0.001) in both groups. By the end of the study, the number of BANA-positive sites dropped to just 20% in Group A and 15% in Group B.

Intergroup Comparison

The Chi-square analysis showed no significant difference in the distribution of BANA status between the groups at three months (p = 0.692) or six months (p = 0.942). The BANA test results are given in Table 2.

**Table 2:** Longitudinal and intragroup analysis of BANA test results Chi-square test (χ^2^ value) (intergroup comparison): Used for comparing the distribution of BANA results (positive, weakly positive, and negative) between Group A and Group B at each specific time point (T0, T3, and T6). Wilcoxon signed-rank test/Friedman test (intragroup comparison): Used for analyzing the significance of the change over time within each group (e.g., baseline vs. three months and baseline vs. six months). McNemar’s test: Used for analyzing the "conversion rate" (e.g., the transition from BANA-positive to BANA-negative) within each group. Significance: (*) denotes statistically significant (p < 0.05); (NS) denotes nonsignificant (p > 0.05). BANA: N-benzoyl-DL-arginine-2-naphthylamide.

Interval	BANA status	Group A (n = 20)	Group B (n = 20)	Intergroup χ^2^	Intergroup p-value	Intragroup significance (vs. baseline)
Baseline (T0)	Positive (p)	16 (80%)	15 (75%)	1.298	0.255 (NS)	—
	Weakly positive (wp)	4 (20%)	5 (25%)			
	Negative (n)	0 (0%)	0 (0%)			
3 months (T3)	Positive (p)	8 (40%)	6 (30%)	0.735	0.692 (NS)	Group A: p < 0.05*; Group B: p < 0.05*
	Weakly positive (wp)	7	10			
	Negative (n)	5	4			
6 months (T6)	Positive (p)	4 (20%)	3 (15%)	0.119	0.942 (NS)	Group A: p < 0.001*; Group B: p < 0.001*
	Weakly positive (wp)	6	7			
	Negative (n)	10	10			

Clinical parameters (GI, PD, and CAL)

All clinical parameters showed a significant longitudinal improvement from baseline to six months in both treatment groups (p < 0.001).

Intergroup Analysis

At baseline, there were no statistically significant differences between Group A (endo only) and Group B (combined) for GI, PD, or CAL (p > 0.05). At the six-month interval, while Group B exhibited numerically superior outcomes specifically in PD (5.10 ± 2.15 mm vs. 5.70 ± 2.11 mm) and CAL (8.10 ± 2.59 mm vs. 8.75 ± 3.26 mm), the differences between the two groups did not reach statistical significance (p = 0.379 and p = 0.488, respectively).

Intragroup Analysis

Both groups demonstrated an early significant improvement from baseline to three months (p < 0.05). However, the F-statistic for the overall study progress was higher in Group B (F = 33.682) compared to Group A (F = 26.845), indicating a stronger healing trend over the six-month period when combined therapy was utilized. The comparison of clinical parameters is shown in Table 3.

**Table 3:** Clinical parameter comparison of gingival index (GI), pocket depth (PD), and clinical attachment level (CAL) Mean ± SD: Results are presented as mean value ± standard deviation. Intergroup comparison: Analyzed using the independent samples t-test (t-statistic) to compare Group A and Group B at specific time points. Intragroup comparison: Analyzed using one-way ANOVA (F-statistic) to evaluate the significance of changes within each group from baseline through six months. Significance: (*) denotes statistically significant (p < 0.05); (NS) denotes nonsignificant (p > 0.05).

Parameters	Interval	Group A	Group B	t-statistic	p-value
Gingival index (GI)	Baseline	2.04 ± 0.39	2.12 ± 0.43	-0.616	0.856NS
	3 months	1.72 ± 0.38	1.53 ± 0.44	1.458	0.153NS
	6 months	1.16 ± 0.41	0.98 ± 0.43	1.389	0.173NS
Pocket depth (PD), mm	Baseline	8.15 ± 3.25	7.95 ± 2.24	0.227	0.821NS
	3 months	6.50 ± 2.52	6.55 ± 2.37	-0.065	0.949NS
	6 months	5.70 ± 2.11	5.10 ± 2.15	0.891	0.379NS
Clinical attachment level (CAL), mm	Baseline	10.65 ± 4.28	10.45 ± 2.87	0.174	0.863NS
	3 months	9.35 ± 3.51	9.30 ± 3.16	0.047	0.962NS
	6 months	8.75 ± 3.26	8.10 ± 2.59	0.700	0.488NS
Intragroup comparison	F-statistic	F = 26.845	F = 33.682	(Significant)	
Baseline to 3 months	p-value	P < 0.05∗	P < 0.05∗		
Baseline to 6 months	p-value	P < 0.001∗	P < 0.001∗		

**Figure 3:**
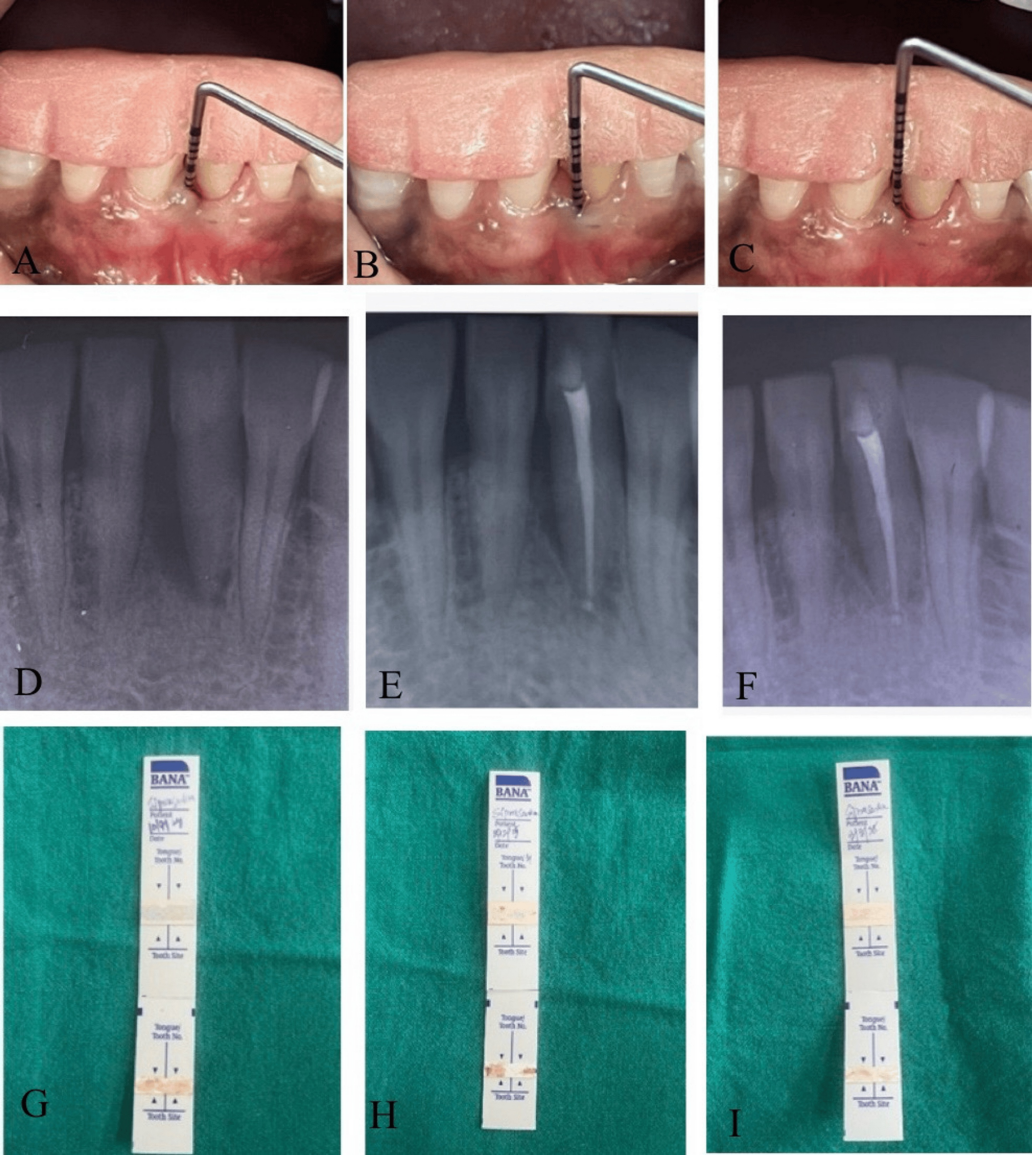
Sequential clinical, radiographic, and enzymatic evaluation of the treatment site at baseline (A, D, G), three-month follow-up (B, E, H), and six-month follow-up (C, F, I) demonstrating progressive reduction in periodontal pocket depth, periapical healing following endodontic and periodontal therapy, and a corresponding decrease in the "red complex" bacterial activity as shown by BANA test strip transitions from positive (dark blue) to negative (salmon) BANA: N-benzoyl-DL-arginine-2-naphthylamide.

Figure 3 demonstrates sequential clinical, radiographic, and enzymatic evaluation of the treatment site from baseline to six-month follow-up following endodontic and periodontal therapy in one of the research participants.

## Discussion

Pulp and periodontal disease are relatively common causes for tooth loss. The intricate relationship between dental pulp and periodontium is referred to as the endodontic-periodontal continuum. Simon et al. (1972) [12] classified the disease as primary endodontic lesion, primary periodontal lesion, primary endodontic lesion with secondary periodontal involvement, primary periodontal lesion with secondary endodontic involvement, and true combined lesion. They present a complex clinical scenario when both tissues are affected by disease. The red complex, a polymicrobial aggregate comprising *P. gingivalis*, *T. forsythia*, and *T. denticola*, exhibits a consistent and strong association with the pathogenesis of periodontal tissue destruction [7,8]. The BANA assay is a rapid chairside diagnostic tool [23,24]. It is employed to detect the presence of proteolytic enzymes, specifically trypsin-like peptidases, produced by *P. gingivalis*, *T. denticola*, and *T. forsythia* (formerly *Bacteroides forsythus*) [25].

In the present study, 40 research participants diagnosed with endo-perio lesions underwent BANA testing at baseline. Remarkably, 30 individuals (75%) exhibited a positive BANA test (>10^5^ colony-forming units (CFU) of red complex bacteria), while the remaining 10 (25%) showed weakly positive results (<10^5^, >10^4^ CFU of red complex bacteria). The high prevalence of BANA-positive outcome aligns with the hypothesis that red complex organisms are heavily implicated in the pathogenesis of endo-perio lesions [7,8]. Mineoka et al. (2008) [21] concluded that co-occurrence of *P. gingivalis*, *T. denticola*, and *T. forsythia* was detected more frequently in subgingival plaque samples from periodontal disease sites (+PD/+BOP-inflamed periodontal pockets) - *P. gingivalis*: 2.7 × 10^4 ^to8.5 ×10^4^, *T. denticola*: 2.3 × 10^2^ to 5.5 × 10^2^, and*T. forsythia*: 2.7 × 10^4^ (±4.5 × 10^4^) - than in those from healthy sites (*P. gingivalis*, *T. denticola*, and *T. forsythia*:* *2.2 × 10^3^ (±1.0 × 10^4^), 7.4 × 10^1^ (±​​​​​​​1.9 × 10^2^), and 2.3 × 10^3^ (±​​​​​​5.5 × 10^3^) mean cell numbers, respectively). ​​​​​​​Thus, a positive BANA (>10^5^ CFU of red complex bacteria) test may serve as a surrogate marker for increased disease severity, greater microbial load, and a potentially more guarded prognosis if not managed appropriately [22,26,27].

The microbiological analysis, using the BANA test as an indicator of the red complex, reveals a more pronounced and statistically significant reduction in BANA-positive bacteria in Group B compared to Group A, particularly at the three-month and six-month evaluations [28]. This suggests that while both treatment approaches led to comparable clinical improvements, they may have had differential effects on the levels of these key periodontal pathogens [12-17]. The slightly more pronounced microbiological effect observed in Group B, despite comparable clinical outcomes, warrants further investigation [18-20]. It is possible that the specific treatment protocol employed in Group B had a more direct or sustained impact on the red complex bacteria, even if the overall clinical benefits were like those of Group A in this six-month time frame. However, the outcomes of this study indicate that endodontic intervention exerts a significant influence on the immediate peri- radicular environment, effectively delaying the sequential progression toward the reconstitution of a complex, mature microbial ecosystem over a six-month observation interval following the endodontic therapy [9-11].

Limitations

The BANA test provides a semiquantitative assessment of the red complex, thus microbiological assessment in this test is limited by not providing a specific identification and quantification of each of the red complex species. Further future research with a larger sample size is needed, employing advanced molecular methodologies and long-term follow-ups, for the specific identification and quantification of red complex bacteria in endodontic-periodontal lesions.

## Conclusions

Within the limits of the present study, the microbiological assessment of endodontic-periodontal lesions offers enhanced insights into the origin of infection and aids in refining diagnostic protocols and treatment planning strategies. The findings of the present study suggest that meticulous endodontic therapy plays a crucial role in modifying the local environment and delaying the re-establishment of a mature microbial community over a six-month post-treatment period. However, it is important to acknowledge the potential for inter-individual variability in treatment response. Specifically, individuals exhibiting a hyperinflammatory phenotype may demonstrate heightened reactivity to lower bacterial loads, potentially leading to localized inflammation that facilitates pathogen regrowth. These findings emphasize the necessity of addressing both the endodontic and periodontal aspects in the management of these complex lesions and highlight the dynamic interaction between clinical and microbiological parameters.

## References

[1] Simring M, Goldberg M (1964). The pulpal pocket approach: retrograde periodontitis. J Periodontol.

[2] Seltzer S, Bender IB, Ziontz M (1963). The interrelationship of pulp and periodontal disease. Oral Surg Oral Med Oral Pathol.

[3] Meng HX (1999). Periodontic-endodontic lesions. Ann Periodontol.

[4] Nandini S (2010). Indirect resin composites. J Conserv Dent.

[5] Cristea M, Eribe ER, Sanz M, Herrera D (2005). Quantitative and qualitative comparison of subgingival and endodontic microbiota in teeth with concomitant periodontal and endodontic lesions. J Clin Periodontol.

[6] Lopes EM, Passini MR, Kishi LT, Chen T, Paster BJ, Gomes BP (2021). Interrelationship between the microbial communities of the root canals and periodontal pockets in combined endodontic-periodontal diseases. Microorganisms.

[7] Suzuki N, Yoneda M, Hirofuji T (2013). Mixed red-complex bacterial infection in periodontitis. Int J Dent.

[8] Socransky SS, Haffajee AD, Cugini MA, Smith C, Kent RL Jr (1998). Microbial complexes in subgingival plaque. J Clin Periodontol.

[9] Rupf S, Kannengiesser S, Merte K, Pfister W, Sigusch B, Eschrich K (2000). Comparison of profiles of key periodontal pathogens in periodontium and endodontium. Endod Dent Traumatol.

[10] Li H, Guan R, Sun J, Hou B (2014). Bacteria community study of combined periodontal-endodontic lesions using denaturing gradient gel electrophoresis and sequencing analysis. J Periodontol.

[11] Gambin DJ, Vitali FC, De Carli JP, Mazzon RR, Gomes BP, Duque TM, Trentin MS (2021). Prevalence of red and orange microbial complexes in endodontic-periodontal lesions: a systematic review and meta-analysis. Clin Oral Investig.

[12] Simon JH, Glick DH, Frank AL (1972). The relationship of endodontic-periodontic lesions. J Periodontol.

[13] Jansson LE, Ehnevid H (1998). The influence of endodontic infection on periodontal status in mandibular molars. J Periodontol.

[14] Tiwari S, Saxena S, Kumari A, Chatterjee S, Hazra A, Choudhary AR (2020). Detection of red complex bacteria, P. gingivalis, T. denticola and T. forsythia in infected root canals and their association with clinical signs and symptoms. J Family Med Prim Care.

[15] Goldman M, Schilder H (1987). Treatment planning for the combined endodontic-periodontal lesion. Dent Clin North Am.

[16] Vakalis KN, Malliarakis NE, Vouros I (2005). Treatment of combined periodontal-endodontic lesions: a case series. J Int Acad Periodontol.

[17] Schmidt JC, Walter C, Amato M, Weiger R (2014). Treatment of periodontal-endodontic lesions--a systematic review. J Clin Periodontol.

[18] Cortellini P, Stalpers G, Mollo A, Tonetti MS (2020). Periodontal regeneration versus extraction and dental implant or prosthetic replacement of teeth severely compromised by attachment loss to the apex: A randomized controlled clinical trial reporting 10-year outcomes, survival analysis and mean cumulative cost of recurrence. J Clin Periodontol.

[19] Tewari S, Sharma G, Tewari S, Mittal S, Bansal S (2018). Effect of immediate periodontal surgical treatment on periodontal healing in combined endodontic-periodontal lesions with communication-a randomized clinical trial. J Oral Biol Craniofac Res.

[20] Gupta S, Tewari S, Tewari S, Mittal S (2015). Effect of time lapse between endodontic and periodontal therapies on the healing of concurrent endodontic-periodontal lesions without communication: a prospective randomized clinical trial. J Endod.

[21] Mineoka T, Awano S, Rikimaru T, Kurata H, Yoshida A, Ansai T, Takehara T (2008). Site-specific development of periodontal disease is associated with increased levels of Porphyromonas gingivalis, Treponema denticola, and Tannerella forsythia in subgingival plaque. J Periodontol.

[22] Muthukumar S, Pushparajan S, Kumar S (2014). Relationship between gingival bleeding and anaerobic periodontal infection assessed by BANA assay in chronic periodontitis patients. J Indian Soc Periodontol.

[23] Loesche WJ, Syed SA, Stoll J (1987). Trypsin-like activity in subgingival plaque. A diagnostic marker for spirochetes and periodontal disease?. J Periodontol.

[24] Loesche WJ, Bretz WA, Kerschensteiner D (1992). Comparison of the BANA test, DNA probes, and immunological reagents for ability to detect anaerobic periodontal infections. J Clin Microbiol.

[25] Constanta C, Mihai A, Didilescu A, Luca R (2006). The clinical importance of BANA test in periodontal disease. Rom J Morphol Embryol.

[26] Grisi MF, Salvador SL, Figueiredo LC (1998). Relationship between clinical probing depth and reactivity to the BANA test of samples of subgingival microbiota from patients with periodontitis. J Periodontol.

[27] Andrade JA, Monteiro L, Ferreira D, Lopes V (2010). Evaluation of the BANA test in detecting different levels of Porphyromonas gingivalis, Treponema denticola, and Tannerella forsythia. Braz Oral Res.

[28] Dhalla N, Patil S, Chaubey KK, Narula IS (2015). The detection of BANA micro-organisms in adult periodontitis before and after scaling and root planing by BANA-Enzymatic™ test kit: an in vivo study. J Indian Soc Periodontol.

